# Uptake of Non-Transferrin Iron by Erythroid Cells

**DOI:** 10.1155/2011/945289

**Published:** 2010-12-13

**Authors:** Eugenia Prus, Eitan Fibach

**Affiliations:** Department of Hematology, Hadassah-Hebrew University Medical Center, Ein-Kerem, P.O. Box 12000, Jerusalem 91120, Israel

## Abstract

Most of the iron in the plasma is bound to transferrin (Tf) and is taken up by cells through their surface Tf receptors (TfRs). Under pathological conditions of iron-overload, the plasma iron which is in excess of the binding capacity of Tf is present as non-Tf-bound iron. We probed the uptake of non-Tf iron and its consequences on the oxidative status of peripheral RBC and reticulocytes as well as developing erythroid precursors grown in vitro. The cells were exposed to ferrous ammonium sulfate under Tf-supplemented and Tf-free conditions. Using flow cytometry techniques, we found that both the TfR-deficient mature RBC and their TfR-containing precursors at all stages of maturation can take up non-Tf iron that accumulates as redox-active labile iron and generates reactive oxygen species. Such a mechanism may account for ineffective erythropoiesis of developing precursors in the bone marrow and for the shortening of the lifespan of mature RBCs in the circulation.

## 1. Introduction

Most of the iron in the plasma is bound to transferrin (Tf) which serves as a carrier protein that mediates the uptake of iron by cells through their surface Tf receptor-1 (TfR) [1]. Intracellularly, iron is released from Tf following a decrease in endosomal pH and is then transported across the endosomal membrane by DMT1 (also known as Nramp2) [2]. Under pathological conditions of iron-overload, plasma iron which is in excess of the binding capacity of Tf is present as non-Tf bound iron (NTBI) [3]. This chemically ill-defined iron and its redox potent labile plasma iron (LPI) can be taken up by cells in vital organs via several pathways and be responsible for the major pathological consequences of iron-overload [4].

In erythroid cells, while most of the iron is in the form of hemoglobin (Hb), some iron is in the form of labile iron [5]. It has a redox potential and generates reactive oxygen species (ROS) which leads to cytotoxic effects [6]. We have previously reported that the labile iron pool (LIP), also termed labile cellular iron (LCI), is increased in RBCs under conditions of iron overload, such as in the case of chronic anemias associated with blood transfusions [7]. Increased LIP in RBCs may be the result of abnormal iron turnover in developing precursors (due to increased uptake from iron-overloaded plasma, diminished utilization because of reduced Hb production, or degradation of unstable Hb). In addition, RBCs may take up NTBI from their environment (the plasma). Iron uptake by RBCs and reticulocytes (retics) has been previously demonstrated in various model systems using radiolabeled iron [8–11]. But, the results have been disputed because the experiments were carried out in artificial media containing high sucrose. In the present study, we probed the uptake of non-Tf iron by various erythroid cells. We utilized a novel flow cytometry methodology [12] to measure the uptake of iron and the generation of ROS by peripheral blood RBCs and retics in their autologous plasma as well as by human developing erythroid precursors grown in vitro. The results showed that both the TfR-deficient mature RBCs and the TfR-bearing erythroid precursors at all stages of maturation can take up non-Tf iron. This uptake was associated with increased LIP and ROS generation. Such a mechanism may account for the hematological consequences of iron-overload—ineffective erythropoiesis and short lifespan of RBCs in the circulation.

## 2. Methods

### 2.1. Blood Cells and Erythroid Cultures

Normal human peripheral RBCs, obtained in heparin-containing tubes, were used in all experiments, unless otherwise stated. Some experiments were performed with RBCs obtained from thalassemic mice. The founders of the mouse colony were obtained from Dr. S. Rivella, Weill Medical College of Cornell University, NY, NY. Heterozygotes (Hbb^th3/+^) mice exhibit severe anemia (7 to 9 g/dL Hb), abnormal RBCs morphology, splenomegaly, and hepatic iron deposition [13]. Animals were bred at the animal facility of Hadassah—Hebrew University Medical Center. The research was approved by the Hadassah—Hebrew University Medical Center Human Experimentation Review Board and the Animal Ethics Committee. All human participants gave written informed consent.

Erythroid cultures were initiated by growing human peripheral blood mononuclear cells in a two-phase liquid culture system as previously described in [14, 15]. In short, cells were first cultured in alpha medium supplemented with 10% fetal calf serum and 10% conditioned medium obtained from cultures of the human 5637 bladder carcinoma cell line and 1 *μ*g/mL cyclosporin A (phase I). After 7 days, nonadherent cells were harvested, washed and suspended in phase II medium, containing alpha medium, 30% fetal calf serum, 1% bovine serum albumin, 10 *μ*M *β*-mercaptoethanol, 1.5 mM glutamine, 10 *μ*M dexamethasone, 5 ng/ml stem cell factor and 1 U/ml human recombinant erythropoietin. Hb-containing cells were scored by staining with benzidine dihydrochloride [15].

### 2.2. Iron and Chelators

Ferrous ammonium sulfate (FAS) (Sigma, St. Louis, MO) was freshly dissolved for each experiment in water to 1 mM. Human holotransferrin (90% saturated) was purchased from Biological Industries (Beit-Hemek, Israel) and added to cells at 300 *μ*g/mL. Deferiprone (L1) (Apotex, Weston, ON, Canada) was freshly dissolved in water to 20 mM and added at 50 *μ*M.

### 2.3. Measurements of Iron Uptake and ROS Generation

Iron uptake was measured as previously described in [12]. For measurement of cytoplasmic LIP cells were loaded for 15 min at 37°C with calcein acetoxymethyl ester (CA-AM) (Sigma-Aldrich, St. Louis, MO) (*1* *μ*M for RBCs and retics, and 0.5 *μ*M for cultured cells). CA-AM enters viable cells and becomes fluorescent upon hydrolysis by esterases; its fluorescence is quenched by binding of LIP [16–19]. Mitochondrial LIP was measured by staining cultured cells with 1 *μ*M rhodamine B-[(1,10-phenanthrolin-5-yl)-aminocarbonyl] benzyl ester (RPA, Squarix biotechnology, Marl, Germany) for 20 min at 37°C as previously described in [12].

ROS were measured by staining with 0.1 mM 2′–7′-dichlorofluorescin diacetate (DCF, Sigma) for 15 min at 37°C [20]. Upon crossing the membrane, this compound undergoes deacetylation by intracellular esterases, producing a nonfluorescent compound that is trapped inside the cells. Its oxidation by ROS produced a highly fluorescent compound—2′–7′-dichlorofluorescine (CA) [21].

### 2.4. Flow Cytometry

Cell fluorescence was analyzed by a flow cytometer (FACS-calibur^R^, Becton-Dickinson, Immunofluorometry systems, Mountain View, CA) as previously described in [12]. A 488-nm argon and 635-nm red diode lasers were used for excitation. At least 20,000 cells were analyzed using logarithmic amplification for the fluorescence signal height and linear amplification for forward light scatter and side light scatter. “Threshold” was set on forward light scatter to exclude cell debris, microparticles and platelets. In some experiments, blood cells were stained simultaneously with CA or DCF and an allophycocyanin-(APC-) conjugated anti-CD71 antibody (Becton-Dickinson, San Jose, CA) for 15 min at 37°C. Cells were gated as CD71^+^ (retics) and CD71^−^ (RBCs), and the green fluorescence (CA or DCF) of each population was measured. Mitochondrial LIP was measured in cultured erythroid cells stained with RPA. The arithmetic Mean Fluorescence Intensities (MFI) were calculated by the CellQuest^R^ software (Becton-Dickinson). For each experiment, unstained cells served as controls; their MFI was <10. The statistical significance was calculated using the two-sample Student's *t*-test for differences in means *P* < .05 was considered significant.

## 3. Results

**Figure 1:**
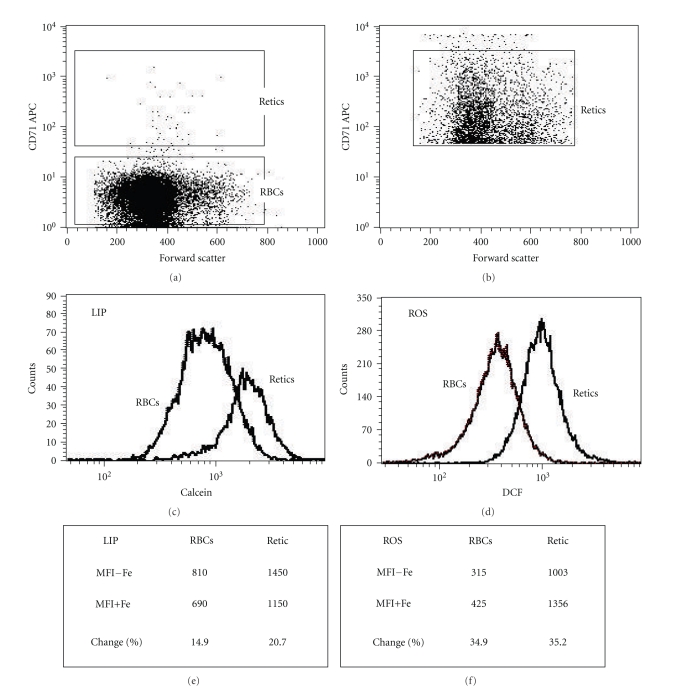
Flow cytometry measurement of iron uptake and reactive oxygen species (ROS) generation in RBCs and reticulocytes. Normal peripheral blood cells were diluted (5 × 10^6^/ml) in PBS, stained with CA-AM or DCF and an APC-conjugated anti-CD71 antibody. Following washing with PBS, they were resuspended in their autologous plasma and then incubated with (+Fe) or without (−Fe) 20 *μ*M FeSO_4_ for 1 hr. (a) A FSC × CD71 dot-plot, showing the gates for RBCs (CD71^−^) and reticulocytes (retics) (CD71^+^). (b) A FSC × CD71 dot-plot of events acquired in the retic gate. (c, d) Histogram distributions of both populations with respect to CA- and DCF-fluorescence, respectively. (e, f) Summaries of the results obtained in each population following incubation with and without Fe. The MFIs and the percent change in the MFIs of each population incubated with and without Fe, calculated per the basal fluorescence (MFI−Fe) are indicated.

The flow cytometry methodology for measuring iron uptake and ROS generation in RBCs and retics is illustrated in Figure 1. Blood cells were diluted in PBS, labeled with an APC-conjugated anti-CD71 antibody and either CA-AM or DCF, washed with PBS, diluted in their autologous plasma, and incubated with or without FAS (20 *μ*M for 1 hr). Figures 1(a) and 1(b) demonstrate FSC × CD71 dot plots of RBCs (CD71^−^) and retics (CD71^+^), respectively. Figures 1(c) and 1(d) show histogram distributions of both populations with respect to CA- and DCF-fluorescence, respectively. Cells incubated without iron showed a much higher basal CA-fluorescence (MFI−Fe 1450 versus 810) and DCF-fluorescence (1003 versus 315) in retics compared to RBCs (Figures 1(e) and 1(f)). Iron uptake resulted in a decrease in the CA-fluorescence (Figure 1(e)) and an increase in the DCF-fluorescence (Figure 1(f)). The data for each population were reported as the percent change in the MFI following incubation with FAS, calculated as percentage of the basal fluorescence [100 × (MFI − Fe – MFI + Fe)/(MFI − Fe)]. In the representative experiment shown in Figure 1, the change in the CA-MFI was 14.9% for RBCs and 20.7% for retics (Figure 1(e)). The change in the DCF-MFI was 34.9% for RBCs and 35.2% for retics (Figure 1(f)).

**Figure 2:**
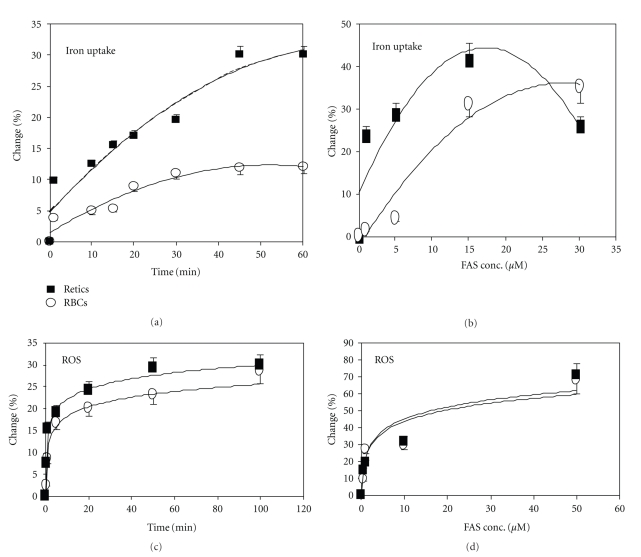
Time- and dose-related iron uptake and ROS generation by RBCs and reticulocytes. Normal peripheral blood cells were labeled, suspended in their autologous plasma and incubated with or without FeSO_4_ (FAS) as described in the legends to Figure 1. (a, c) Labeled cells were incubated with or without 20 *μ*M FAS for the indicated durations. (b, d) Labeled cells were incubated with the indicated concentrations of FAS for 1 hr. The results of iron uptake (a, b) and ROS generation (c, d) in RBCs (∘) and retics (■) are expressed as the percent (%) change (mean ± SD, *N* = 4) in the MFIs following incubation with and without Fe, calculated per the MFI−Fe.

Using this methodology, we studied the time- and dose-related effects (Figures 2(a) and 2(b), resp.) of iron uptake by RBCs and retics. Iron uptake was detected as early as 1 min after addition of FAS. It was faster in retics than in RBCs; in RBCs it plateaued after 45 min, whereas in retics it continued to increase for 3 hrs, reaching 6.8-fold higher levels than in RBCs. In both RBCs and retics, iron uptake was dose dependent; it was detected at 1 *μ*M FAS and increased up to 20 *μ*M. Iron uptake was associated with increased ROS generation during the first 15–30 min in both RBCs and in retics (Figures 2(c) and 2(d)).

Normal plasma contains unsaturated Tf, therefore some of the iron added in these experiments became bound to Tf. Since mature RBCs are devoid of TfR [22], their iron uptake is necessarily Tf independent. To substantiate this point and to determine whether such a mechanism is operative also in retics, iron uptake was measured in parallel in Tf-containing (autologous plasma) and Tf-free media. The results showed that iron uptake was slower in plasma (2.5-fold in RBCs and 2-fold in retics, both *P* < .01) and indicated that both cells take up iron by a Tf-independent mechanism.

In order to probe the nature of the incoming iron, CA-loaded RBCs were exposed to 20 *μ*M FAS in PBS, washed, and then further incubated with or without the cell-permeable iron-chelator-L1 (50 *μ*M for 1 hr). The results showed a 33% (*P* = .005) increase in the CA-MFI following incubation with L1, similar to the fluorescence of iron-untreated cells. The ability of L1 to overcome the iron-mediated quenching of CA, most probably by binding and thus removing iron from its complex with CA, fulfils the operational definition of the incoming iron as LIP [12].

To establish the pathological significance of non-Tf iron uptake, we used blood from heterozygotes (Hbb^th3/+^) mice, a mouse model of *β*-thalassemia, which suffer from iron-overload [13]. CA-loaded normal mouse RBCs were incubated for 1 hour in plasma derived either from normal or thalassemic mice. The results showed lower CA-MFI of RBCs incubated in thalassemic plasma by 10 ± 2% (*P* = .005), indicating increased LIP than the same RBCs incubated in normal plasma. These results suggest that in thalassemia, continuous, Tf-independent, uptake of iron from the plasma might have a significant effect on the RBCs LIP content.

**Figure 3:**
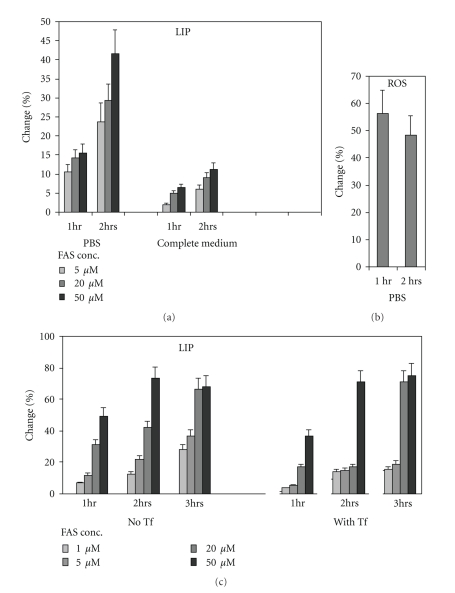
The effect of transferrin (Tf) on iron uptake and ROS by cultured erythroid precursors. Cultured human erythroid precursors were harvested on days 7-8 of phase II, washed and loaded with CA or DCF. (a) CA-loaded cells were washed and resuspended in PBS or in phase II medium (complete medium, containing 30% serum), and incubated for 1- or 2 hrs with the indicated concentrations of FAS. (b) DCF-loaded cells were washed and resuspended in PBS, and incubated for 1- or 2 hrs with the indicated concentrations of FAS. (c) CA-loaded cells were washed and resuspended in PBS supplemented with or without human holo-Tf (300 *μ*g/mL) and incubated for 1–3 hrs with the indicated concentrations of FAS. The results indicated reduced iron uptake in the presence of Tf. The results are expressed as the percent (%) change (mean ± SD, *N* = 4) in the MFIs following incubation with and without Fe, calculated per the MFI−Fe.

To determine the ability of developing erythroid precursors to take up iron in a Tf-independent pathway, cultured erythroid precursors were harvested on days 7-8 of phase II cultures, loaded with CA, washed, and resuspended in phase II medium (containing 30% serum) or in PBS and incubated for 1 or 2 hrs with different concentrations of FAS. The results (Figure 3(a)) indicated enhanced iron uptake in PBS than in serum-containing medium (*P* = .001). Tf-independent iron uptake in PBS was associated with increased ROS generation as reflected by DCF fluorescence (Figure 3(b)). In another set of experiments, the CA-loaded cells were washed, and resuspended in PBS supplemented with or without Tf (300 *μ*g/mL) and incubated for 1–3 hrs with different concentrations of FAS (Figure 3(c)). The results showed that in the presence of Tf, iron uptake was slower, but after 3 hrs, at the higher concentrations of FAS (20–50 *μ*M), the intracellular iron reached a comparable level to that of cells incubated without Tf.

**Figure 4:**
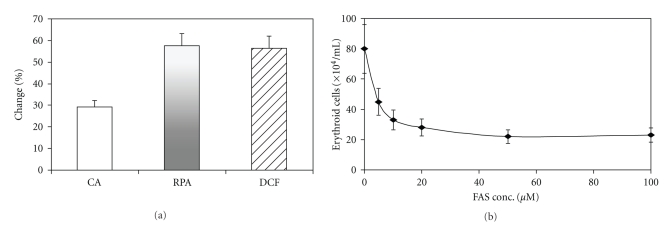
The effects of non-transferrin iron uptake on cytoplasmic and mitochondrial LIP and ROS generation by cultured erythroid precursors. Cultured human erythroid precursors were harvested on day 6 of phase II, incubated with or without 20 *μ*M FAS for 1 hr, and stained with CA, RPA or DCF for measurement of cytoplasmic and mitochondrial LIPs and ROS generation, respectively. The results are expressed as the percent (%) change (mean ± SD, *N* = 4) in the MFIs following incubation with and without Fe, calculated per the MFI−Fe. (b) The indicated concentrations of FAS were added to day 6 cultures. Hb-containing cells were counted by benzidine staining on day 9. The results are expressed as the (mean ± SD, *N* = 4).

When erythroid precursors from day 6 phase II cultures were washed and incubated in PBS with 20 *μ*M FAS for 1 hr, they demonstrated not only an increased cytoplasmic LIP but also a significant increase (*P* = .001) in mitochondrial LIP, assayed by the change in the fluorescence of a mitochondrial-specific probe (RPA), and in ROS (Figure 4(a)).

The effect of non-Tf iron on development of erythroid cells was demonstrated by adding various concentrations of FAS to day 6 phase II cultures. Counting Hb-containing cells after 3 days indicated a significant decrease (FAS = 100 *μ*M versus FAS = 0, *P* < .001) by the addition of iron (Figure 4(b)).

## 4. Discussion

The labile iron pool (LIP) is considered the main cause of malfunctioning of vital organs (e.g., heart, liver, endocrine glands) of patients with iron-overload [3, 23]. Increased LIP may be deleterious to erythroid cells as well; it may be involved in increased apoptosis of developing precursors in the bone marrow (ineffective erythropoiesis) [24] and in shortening of the lifespan of mature RBCs due to extravascular hemolysis. The cytotoxic effects of LIP are probably mediated by increased generation of ROS and the resultant state of oxidative stress [25, 26]. In thalassemia, where iron overload is caused by enhanced iron absorption in the gastrointestinal track [27] and by frequent blood transfusions, we have shown that LIP is high in RBCs and retics, as well as in immature developing erythroid precursors, compared to their normal counterparts [7].

Increased LIP in RBCs may be the result of leftover of unused iron in precursors due to increased uptake (from iron-overloaded plasma), diminished utilization because of reduced Hb production, or degradation of unstable Hb. In addition, RBCs may take up iron from their environment, especially when the amount of iron in the plasma exceeds the binding capacity of Tf. Since mature RBCs are devoid of TfR [22], this uptake is necessarily of non-Tf iron.

In the present study we probed the uptake of non-Tf iron supplied as FAS and its consequences on ROS generation in normal human peripheral blood RBCs and retics as well as in their precursors grown in vitro. The incoming iron and the ROS generation were measured by flow cytometry of cells loaded with fluorescent probes, CA or DCF, respectively. Iron uptake resulted in a decrease in CA-fluorescence while ROS generation resulted in an increase in DCF-fluorescence. The results were expressed as the percent change in the MFI of iron-treated cells compared to untreated cells. Dual staining with CA or DCF and APC-conjugated antibodies to TfR (CD71) permitted simultaneous analysis of RBCs (TfR-negative cells) and retics (TfR-bearing cells). The results indicated that iron uptake by RBCs and retics was dose-and time dependent. It was detected at FAS concentrations as low as 1 *μ*M and increased at higher concentrations. The uptake was faster and reached higher levels in retics than in RBCs. The ability of the incoming iron to bind and quench the fluorescence of CA indicated its labile, chelatable nature. Indeed, when the potent, cell permeable iron-chelator, deferiprone (L1), was added to FAS-treated cells it increased the CA-fluorescence to levels of FAS-untreated cells.

The results also showed that iron uptake by both cell types was associated with an enhanced ROS generation, which was ameliorated by L1. The latter results are in agreement with our previous report that in vitro treatment of iron-overloaded thalassemic RBCs with clinically used iron-chelators, including L1, decreased their LIP as well as ROS [28].

In the present study, iron uptake was first analyzed by exposing peripheral blood cells to FAS in their autologous plasma. Normal plasma, however, contains unsaturated Tf; some of the added iron in these experiments could, therefore, bind to and delivered by Tf. Since mature RBCs are devoid of TfR [22], their iron uptake was necessarily Tf independent. To study whether Tf-independent iron uptake was also operative in TfR-carrying retics, we exposed the cells to FAS in parallel in Tf-containing (autologous plasma) and in Tf-free (PBS) media. The results showed slower iron uptake in plasma than in PBS and indicated those both RBCs and retics take up iron by Tf-independent mechanisms. These results are in agreement with previous data that normal mouse retics acquire non-Tf iron at rates much higher than that at which iron is taken up physiologically from Tf [29]. These results and our present data suggest that in iron-overload, when non-Tf iron is present in the serum, the levels of Tf-receptors do not represent a limiting factor in iron uptake.

The uptake of non-Tf iron by rats and mouse RBCs and retics has been previously studied in vitro using radio-labeled iron. Under the experimental conditions used (e.g., high sucrose-containing medium), it was found that immature erythroid cells acquire non-Tf iron, most probably through their DMT1 transmembrane transport system. But, since physiologically all iron in the circulation is Tf-bound, DMT1 expressed at the plasma membrane has no substrate; it was assumed to function not as an external transporter but to mobilize iron intracellularly, for example, iron transport across the endosomal or the mitochondrial membranes [29].

The uptake of non-Tf iron by developing erythroid cells was studied in cultures derived from normal human erythroid progenitors. These cultures have been previously demonstrated to recapitulate erythropoiesis in vivo, including various aspects of iron metabolism [30]. Following culture in serum-containing medium, the cells were harvested, washed, loaded with CA, and resuspended in PBS or complete serum-containing culture medium. Iron entry was faster and to a larger extent in the absence of serum indicating that Tf-independent iron uptake is operative in these cells too. We then studied the direct effect of Tf. The results indicated that iron uptake from Tf was much slower than that from FAS. When holo-Tf (90% saturated) was added together with FAS, iron uptake was retarded compared to FAS alone. It is interesting to note in this context that treating thalassemic mice with apo-Tf has been shown to reduce their iron load [31].

The results indicated that RBCs, retics as well as erythroid precursors at early stages of maturation take up iron through a Tf-independent pathway. It is unlikely that this pathway is operative normally, but only under pathological iron-overload situation when NTBI appears in the serum. Whether erythroid cells at various stages of maturation share the same mechanism of Tf-independent iron uptake is still an open question. Although the incoming non-Tf iron is found in the mitochondria of erythroid precursors, as determined by reducing the RPA fluorescence (Figure 4), it is not likely to participate in heme synthesis and Hb production. We have previously shown that, unlike Tf-iron and to some extent iron-ferritin and hemin, non-Tf iron cannot support the survival, proliferation, and hemoglobinization of erythroid precursors in vitro [32]. In contrast, non-Tf iron uptake involves in the induction of ROS generation and its cytopathological consequences, as demonstrated by a decrease in erythroid cell yield in cultures exposed to FAS (Figure 4).

Finally, our results suggest that flow cytometry analyses of LIP and ROS in RBCs might reflect “real-time” iron accumulation and as such might complement the measurements of serum ferritin—an acute phase reactant which is elevated in various inflammatory states, not necessarily related to iron overload [33]. The LIP assay does not reflect iron that has already accumulated in tissues and therefore cannot replace liver biopsy or MRI; however, since this assay is nonhazardous and much cheaper, when performed on multiple occasions, it might predict potential iron overload in tissues as well.

## 5. Conclusions

RBCs, retics, and developing erythroid precursors take up iron through a Tf-independent pathway. This pathway is operative under pathological iron-overload situation in the presence of non-Tf iron in the serum. The incoming non-Tf iron does not participate in heme synthesis and Hb production, but induces ROS generation, which results in cytotoxicity and a decrease in the erythroid cell yield. In addition, the flow cytometry methodology used in the present study may provide an analytical platform for practical and accurate measurements of LIP and ROS in RBCs for evaluating iron-overload and chelation therapy. 

##  Authors Contribution

E. Prus performed the research, analyzed data and participated in writing the paper. E. Fibach designed the research, analyzed data and wrote the paper. 

##  Conflict of Interests

The authors declare no competing financial interests.
